# Control of yellow and purple nutsedge in elevated CO_2_ environments with glyphosate and halosulfuron

**DOI:** 10.3389/fpls.2015.00001

**Published:** 2015-01-20

**Authors:** S. Christopher Marble, Stephen A. Prior, G. Brett Runion, H. Allen Torbert

**Affiliations:** ^1^Department of Environmental Horticulture, Mid-Florida Research and Education Center, Institute of Food and Agricultural Sciences, University of FloridaApopka, FL, USA; ^2^Agricultural Research Service, National Soil Dynamics Laboratory, U.S. Department of AgricultureAuburn, AL, USA

**Keywords:** climate change, invasive species, herbicide, nutsedge, resistance management

## Abstract

Atmospheric concentrations of carbon dioxide (CO_2_) have significantly increased over the past century and are expected to continue rising in the future. While elevated levels of CO_2_ will likely result in higher crop yields, weed growth is also highly likely to increase, which could increase the incidence of herbicide resistant biotypes. An experiment was conducted in 2012 to determine the effects of an elevated CO_2_ environment on glyphosate and halosulfuron efficacy for postemergence control of purple and yellow nutsedge (*Cyperus rotundus* L. and *C. esculentus* L.). Both species of nutsedge where grown in 3.0-L containers under either ambient or elevated (ambient + 200 μmol mol^−1^) CO_2_ in open-top field chambers and treated with either 0.5×, 1.0×, or 1.5× of the manufacturer's labeled rate of halosulfuron, glyphosate, or a tank mix of the two herbicides. The growth of both nutsedge species responded positively to elevated CO_2_, purple nutsedge had increased shoot and root dry weights and yellow nutsedge had increased shoot, root, and tuber dry weights and counts. Few treatment differences were observed among the herbicides at any of the rates tested. At 3 weeks following herbicide application, both purple and yellow nutsedge were adequately controlled by both herbicides and combinations at all rates tested, regardless of CO_2_ concentration. Based on this study, it is likely that predicted future CO_2_ levels will have little impact on the efficacy of single applications of halosulfuron or glyphosate for control of purple and yellow nutsedge at the growth stages described here, although scenarios demanding more persistent control efforts remain a question.

## Introduction

Atmospheric concentrations of CO_2_ have been steadily rising each year, from approximately 315 ppm in 1958 to 385 ppm in 2009 (Keeling et al., 2009). Carbon dioxide levels are expected to continue increasing in the future, with some estimates showing an increase from the current level of ~380 ppm to 700 ppm by year 2100 (IPCC, 2007). Increases in CO_2_ and other greenhouse gasses are largely attributed to anthropogenic causes such as land use changes and fossil fuel combustion, both widely indicated as primary causes of global warming (IPCC, 2007). The potential impact of climate change on agricultural production has become a critical issue that deserves investigation in order to determine the best methods of adapting current production practices to meet the demands of a changing environment (Eitzinger et al., 2010; Calanca et al., 2011).

It is well established that elevated CO_2_ increases growth and yield of most plant species (Patterson and Flint, 1980; Kimball, 1983), primarily attributable to increased rates of photosynthesis and water use efficiency (Rogers and Dahlman, 1993; Amthor, 1995). Weeds represent a major constraint on crop production (Oerke, 2006) and competition dynamics between weed and crop plants have been shown to change with CO_2_ enrichment (Patterson and Flint, 1980). Plants that utilize the C_3_ photosynthetic pathway are predicted to benefit the most from increased atmospheric levels of CO_2_ because the current CO_2_ concentration is suboptimal for C_3_ photosynthesis (Leegood, 2002) and additional CO_2_ can cause a “fertilization” effect, resulting in higher growth rates (Ziska, 2002). Carbon dioxide fixation has been shown to be saturated at 360 ppm for C_4_ plants, making them less likely to show an increased growth response when exposed to elevated levels of CO_2_ (Leegood, 2002). As most of the world's crop species are C_3_ plants and many of the major weed species are C_4_ plants, these changes in growth, or lack thereof, in elevated CO_2_ environments suggest that crops could gain a competitive advantage over many of the major weed species under elevated CO_2_. In reality this is not likely the case (Ziska et al., 1999); other factors such as changes in herbicide efficacy and the ability of weed species to outcompete crop species may come into play and limit this advantage (Archambault et al., 2001). Weed species are also more likely to have greater genetic diversity and physiological plasticity when compared to crop species, and therefore are more likely to be able to adapt to a changing environment (Ziska and Runion, 2007).

Environmental factors such as temperature, precipitation and relative humidity influence the efficacy of herbicides (Archambault et al., 2001). Additionally, there have been several studies that report decreased herbicide efficacy for control of numerous annual and perennial weeds in elevated CO_2_ environments (Archambault et al., 2001; Ziska and Runion, 2007). Plants that are grown in CO_2_-enriched environments often develop high concentrations of starch in leaves (Patterson, 1995; Ziska et al., 1999) and greater total leaf area and biomass (Ziska et al., 2007) which can cause a dilution effect on herbicides, rendering them less effective at current labeled rates (Manea et al., 2011). Additionally, C_3_ plants have been shown to have decreased stomatal conductance and increased leaf thickness in elevated CO_2_ which may also limit foliar uptake of herbicides (Ziska and Teasdale, 2000; Ainsworth and Long, 2005).

Determining how weed species will respond to increased levels of CO_2_, and subsequently the effect of increased CO_2_ on herbicide efficacy on these weeds is critical. Two of the world's most prevalent weeds are purple nutsedge (*Cyperus rotundus* L.) and yellow nutsedge (*C. esculentus* L.), both of which are perennial C_4_ sedges (Santos et al., 1997). Both purple and yellow nutsedge are difficult to control due to prolific reproduction by underground tubers and seeds (Thullen and Keeley, 1979; Wills, 1987) and because cultivation often increases infestation. Purple nutsedge is native to Eurasia (USDA-ARS, 1970), infests 52 crops in over 90 countries and has been ranked as the “world's worst weed” (Holm et al., 1991a,b). Yellow nutsedge is native to the Eastern Mediterranean (Steckel, 2007), is found on every continent with the exception of Antarctica, and has been ranked as the 16th worst weed in the world (Holm et al., 1991a,b).

Currently, purple and yellow nutsedge are primarily controlled by means of post-emergent herbicide applications. Two of the more common active ingredients used to control nutsedge species include halosulfuron and glyphosate, both of which are labeled to control yellow and purple nutsedge as a directed or over-the-top spray in a variety of crops (Anonymous, 2002, 2011). As herbicide efficacy has been shown to be affected by elevated CO_2_ (Patterson and Flint, 1980, 1990; Ziska et al., 1999; Manea et al., 2011) and both yellow and purple nutsedge growth has been shown to increase in CO_2_ enriched environments (Rogers et al., 2008), the objective of this study was to determine if the efficacy of a single application of glyphosate and halosulfuron for control of yellow and purple nutsedge would be effected in an elevated CO_2_ environment.

## Materials and methods

Control of purple (*Cyperus rotundus* L.) and yellow (*C. esculentus* L.) nutsedge in a CO_2_-enriched environment was evaluated in 2012. Herbicides tested include glyphosate (RoundUp® Pro, Monsanto, St. Louis, MO. 63167, USA) and halosulfuron (SedgeHammer™ 75DF, Gowan, Yuma, AZ. 85364, USA). Glyphosate and halosulfuron were selected based upon availability, widespread use, and previous reports showing effective nutsedge control at label rates (McCloskey, 2004; Felix and Newberry, 2012).

On June 8, 300 nursery pots (3.0-L) were filled with Faford® 2B potting mix (SunGro® Horticulture, Agawam, MA. 01001, USA) and placed in full sun in a gravel area near the experiment site (outside of CO_2_ chambers) and watered in by hand. On June 12, six nutsedge tubers (Azlin Seed Service, Leland, MS) of either yellow or purple nutsedge were planted 2.5 cm below the media surface in 150 pots each (25% more pots than were needed for the experiment were planted with nutsedge tubers from each species to ensure plants could be selected for uniformity of growth at the time of herbicide application). Each pot was then fertilized by topdressing using 17N-2.2P-9.1K Polyon (Harrell's Fertilizer Inc., Sylacauga, AL) control-release fertilizer (10–12 month) at a rate of 20 g (8.31 kg/m^3^) per pot. Tubers from both species began to germinate on June 17 and 18. On June 18, 120 pots of each species were selected for uniformity of growth and tuber germination and placed inside either ambient- or elevated-level CO_2_ chambers. A total of 12 outdoor open-top chambers in full sun (Rogers et al., 1983) with a gravel floor covering 7.3 m^2^ of ground surface area were used to expose plants to ambient and elevated (ambient + 200 μmol mol^−1^) CO_2_ concentrations using a system previously described by Mitchell et al. (1995). Actual daytime CO_2_ concentrations (± standard error) for the duration of the study were 405.6 ± 0.2 μmol mol^−1^ (ambient; 6 chambers) and 608.0 ± 0.6 μmol mol^−1^ (elevated; 6 chambers). Ten pots of each nutsedge species were placed inside each of the 12 chambers, resulting in a total of 60 pots of yellow and purple nutsedge inside ambient chambers (10 pots of each species inside each of the 6 chambers) and 60 pots of yellow and purple nutsedge inside elevated chambers (10 pots of each species inside each of the 6 chambers). Pots were grouped by species and arranged randomly near the center of each chamber. Nutsedge remained inside the chambers for 16 days growing to approximately 10 and 20 cm in height for purple and yellow nutsedge, respectively.

On July 3 (scattered clouds, 32 C, 52% relative humidity, winds NNW at 8 kph), all pots were removed from the chambers and herbicide treatments (Table 1) were applied at an application rate of 187 liters/ha using a CO_2_ backpack sprayer with an 8004 flat fan nozzle (TeeJet Technologies, Wheaton, IL. 60187, USA) at 172.4 kPa. When halosulfuron was applied alone, a nonionic surfactant (Activator 90; Loveland Products, Greeley, CO. 80632, USA) was added at 0.25%. On July 4, pots were placed back into the chambers (either ambient or elevated CO_2_) in the same manner as previously described.

**Table 1:** **Effects of ambient (*A* = 405.6 ± 0.2 μmol mol^−1^) and elevated (*E* = 608 ± 0.6 μmol mol^−1^) CO_2_ levels on purple and yellow nutsedge control ratings following applications of halosulfuron, glyphosate, or a tank mix combination of the two herbicides**.

		**Control ratings**^a^								
**Treatment^b^**		**1 WPT^c^**			**2 WPT**			**3 WPT**		
**Herbicide**	**Rate (kg ai/ha^−1^)**	**A**	**E**	***P*^d^**	**A**	**E**	***P***	**A**	**E**	***P***
**PURPLE NUTSEDGE**										
Halosulfuron	0.04 (0.5×)	3.7 d^e^	3.5 d	0.6700	9.5 ab	8.5 c	**0.0024**	10.0 a	10.0 a	1.0000
Halosulfuron	0.07 (1.0×)	4.0 d	4.3 cd	0.4367	9.8 ab	9.3 abc	0.1230	10.0 a	10.0 a	1.0000
Halosulfuron	0.10 (1.5×)	4.3 d	4.3 cd	1.0000	9.8 ab	9.8 ab	1.0000	10.0 a	10.0 a	1.0000
Glyphosate	1.68 (0.5×)	6.2 c	5.5 bc	0.1215	9.6 ab	8.7 bc	**0.0024**	10.0 a	10.0 a	1.0000
Glyphosate	3.36 (1.0×)	7.8 ab	7.7 a	0.6970	9.0 b	9.2 abc	0.6053	10.0 a	9.8 a	0.1170
Glyphosate	5.04 (1.5×)	7.7 ab	8.0 a	0.4367	9.8 ab	10.0 a	0.6053	10.0 a	10.0 a	1.0000
Combination	0.04 + 1.68 (0.5×)	7.0 bc	6.8 ab	0.6970	9.8 ab	9.8 ab	1.0000	9.8 a	9.8 a	1.0000
Combination	0.07 + 3.36 (1.0×)	8.2 ab	7.8 a	0.4367	9.8 ab	9.7 abc	0.1170	10.0 a	9.8 a	0.1170
Combination	0.10 + 5.04 (1.5×)	8.5 a	8.0 a	0.2443	10.0 a	10.0 a	1.0000	10.0 a	10.0 a	1.0000
Non-treated	0.0	0.0 e	0.0 e	1.0000	0.0 c	0.0 d	1.0000	0.0 b	0.0 b	1.0000
**YELLOW NUTSEDGE**										
Halosulfuron	0.04 (0.5×)	5.2 b	4.7 d	0.2126	9.3 a	8.7 c	**0.0069**	9.8 a	9.5 a	0.1193
Halosulfuron	0.07 (1.0×)	5.7 b	5.7 cd	1.0000	9.7 a	9.0 bc	**0.0069**	10.0 a	10.0 a	1.0000
Halosulfuron	0.10 (1.5×)	5.8 b	6.3 bc	0.2126	9.5 a	9.5 ab	1.0000	10.0 a	10.0 a	1.0000
Glyphosate	1.68 (0.5×)	6.3 b	6.2 c	0.6768	9.5 a	9.5 ab	1.0000	10.0 a	9.3 a	**0.0204**
Glyphosate	3.36 (1.0×)	8.0 a	8.3 a	0.4050	10.0 a	9.5 ab	**0.0410**	9.8 a	9.3 a	**0.0204**
Glyphosate	5.04 (1.5×)	8.2 a	7.6 ab	0.2126	10.0 a	10.0 a	1.0000	9.8 a	9.8 a	0.4339
Combination	0.04 + 1.68 (0.5×)	8.0 a	8.3 a	0.4050	10.0 a	10.0 a	1.0000	10.0 a	10.0 a	1.0000
Combination	0.07 + 3.36 (1.0×)	8.7 a	8.5 a	0.6768	10.0 a	10.0 a	1.0000	10.0 a	10.0 a	1.0000
Combination	0.10 + 5.04 (1.5×)	8.7 a	8.5 a	0.6768	10.0 a	10.0 a	1.0000	10.0 a	10.0 a	1.0000
Non-treated	0.0	0.0 c	0.0 e	1.0000	0.0 b	0.0 d	1.0000	0.0 b	0.0 b	1.0000

a *Control ratings were taken on a scale of 0 to 10, 0 = no control, 10 = complete plant death*.

b *Combination = halosulfuron + glyphosate at the specified rates. Rate relative to manufacturer's recommended rate shown parenthetically*.

c *WPT, weeks post-treatment*.

d *P-values show main effects of CO_2_ level within each treatment on each data collection date. P-values are bolded where CO_2_ had a significant effect on control ratings within a treatment*.

e *Means followed by the same letter within each column and subheading are not significantly different based on Tukey's Honest Significance Difference Test (P ≤ 0.05)*.

The study was designed as a randomized complete block design; the site was divided into six blocks with blocks occurring along the length of the test site and each CO_2_ treatment being randomly assigned to one open-top chamber within each block prior to study initiation. Each herbicide treatment was replicated 10 times in each CO_2_ treatment for both purple and yellow nutsedge in each of the 12 chambers. A non-treated control group was also included for both species inside each of the 12 chambers. Nutsedge control ratings were visually estimated using general nutsedge vigor taking into account plant color, structure, and any foliage abnormalities in the scale (scale of 0 to 10, 0 = no control, green foliage, upright growth, no abnormalities; 10 = complete death, dark brown foliage with no presence of green foliage remaining, no discernable structure, plants lying flat on soil surface) at 1, 2, and 3 weeks post-treatment. At 3 weeks post-treatment, all nutsedge were destructively harvested. Shoots were cut at the soil surface and roots were separated from tubers and washed to remove any remaining debris. Tubers were counted and then washed similarly to roots. Following the destructive harvest, shoots, roots and tubers were dried in a forced air oven at 55 C for 7 days at which time dry weights (DW) were recorded. Data were analyzed using the general linear models procedure (Proc GLM) of SAS (Littell et al., 1996) and treatment comparisons were made using Tukey's Honest Significance Difference Test. In all cases, differences and main effects were considered significant at *P* ≤ 0.05.

## Results

### Purple nutsedge

At 1 week post-treatment, all treatments containing glyphosate generally had higher control ratings than treatments containing halosulfuron alone in both ambient and elevated CO_2_ chambers (Table 1). Data also showed no main effect for CO_2_ at 1 week post-treatment. By 2 weeks post-treatment, few differences were observed among treatments with control ratings ranging from 8.5 (halosulfuron at 0.04 kg ai/ha^−1^) to 10.0 (halosulfuron + glyphosate at 0.10 and 5.04 kg ai/ha^−1^). However, at this time control ratings were higher in ambient chambers compared with elevated chambers for purple nutsedge treated with the lowest rate of halosulfuron and the lowest rate of glyphosate. Almost all treatments provided complete control of purple nutsedge at 3 weeks post-treatment, and no differences were observed among treatments or CO_2_ levels.

Shoot and root DW show that purple nutsedge grew larger in elevated CO_2_ chambers compared with those grown in ambient chambers (*P* = 0.0001) (Table 2). While purple nutsedge grew larger in elevated chambers, no differences were observed in shoot or root DW for purple nutsedge treated with herbicide. Tuber DW and counts were similar in both ambient and elevated chambers and were generally similar among all treatments.

**Table 2:** **Effects of ambient (*A* = 405.6 ± 0.2 μmol mol^−1^) and elevated (*E* = 608 ± 0.6 μmol mol^−1^) CO_2_ levels on purple and yellow nutsedge dry weights and tuber counts following applications of halosulfuron, glyphosate, or a tank mix combination of the two herbicides**.

**Treatment**^a^		**Shoot Dry Wt. (g)**			**Root Dry Wt. (g)**			**Tuber Dry Wt. (g)**			**Tuber Count**		
**Herbicide**	**Rate (kg ai/ha^−1^)**	**A**	**E**	***P*^b^**	**A**	**E**	***P***	**A**	**E**	***P***	**A**	**E**	***P***
**PURPLE NUTSEDGE**													
Halosulfuron	0.04 (0.5×)	1.6 b^c^	1.8 b	0.5821	0.6 b	0.6 b	0.8172	1.1 a	0.9 a	0.6495	12.2 ab	12.3 a	0.9387
Halosulfuron	0.07 (1.0×)	1.9 b	2.1 b	0.5483	0.6 b	0.7 b	0.8897	1.1 a	1.2 a	0.5164	13.3 a	14.8 a	0.4893
Halosulfuron	0.10 (1.5×)	1.7 b	2.1 b	0.2125	0.6 b	0.6 b	0.8897	0.9 a	1.1 a	0.3993	12.3 ab	13.3 a	0.6447
Glyphosate	1.68 (0.5×)	1.5 b	2.0 b	0.1114	0.3 b	0.4 b	0.7463	0.8 a	1.2 a	0.0936	10.5 ab	11.3 a	0.7007
Glyphosate	3.36 (1.0×)	1.3 b	1.8 b	0.1486	0.3 b	0.3 b	1.0000	1.0 a	1.3 a	0.2439	10.3 ab	11.8 a	0.4893
Glyphosate	5.04 (1.5×)	1.6 b	2.0 b	0.2944	0.4 b	0.3 b	0.9263	0.8 a	0.9 a	0.6969	9.5 ab	10.7 a	0.5906
Combination	0.04 + 1.68 (0.5×)	1.6 b	2.2 b	0.1114	0.3 b	0.5 b	0.5793	0.9 a	1.0 a	0.6969	10.7 ab	10.2 a	0.8176
Combination	0.07 + 3.36 (1.0×)	1.4 b	1.8 b	0.2944	0.3 b	0.4 b	0.7116	0.6 a	0.9 a	0.2439	8.0 b	9.7 a	0.4425
Combination	0.10 + 5.04 (1.5×)	1.4 b	1.8 b	0.2125	0.4 b	0.4 b	0.8897	0.7 a	0.8 a	0.6495	8.3 b	10.3 a	0.3571
Non-treated	0.0	11.1 a	14.1 a	**0.0001**	10.6 a	13.3 a	**0.0001**	1.2 a	1.1 a	0.7454	11.0 ab	13.8 a	0.1930
**YELLOW NUTSEDGE**													
Halosulfuron	0.04 (0.5×)	3.8 b	3.8 b	0.9720	0.9 b	0.8 b	0.8326	0.6 ab	0.7 b	0.6768	5.3 ab	5.7 b	0.8514
Halosulfuron	0.07 (1.0×)	3.5 b	4.1 b	0.5393	0.7 b	0.9 b	0.7916	0.6 ab	0.6 b	0.9170	6.0 ab	5.7 b	0.8514
Halosulfuron	0.10 (1.5×)	3.8 b	4.1 b	0.7521	0.8 b	0.8 b	0.9789	0.5 ab	0.6 b	0.4051	4.7 ab	5.0 b	0.8514
Glyphosate	1.68 (0.5×)	3.9 b	4.0 b	0.8883	0.6 b	0.6 b	1.0000	0.6 ab	0.6 b	0.9170	5.3 ab	5.5 b	0.9254
Glyphosate	3.36 (1.0×)	3.0 b	3.3 b	0.7521	0.7 b	0.6 b	0.9158	0.3 b	0.5 b	0.1465	3.3 b	5.7 b	0.1915
Glyphosate	5.04 (1.5×)	3.2 b	3.7 b	0.6232	0.6 b	0.6 b	0.9786	0.4 ab	0.4 b	0.9170	4.3 b	5.3 b	0.5743
Combination	0.04 + 1.68 (0.5×)	3.6 b	3.4 b	0.8686	0.7 b	0.5 b	0.7512	0.6 ab	0.6 b	0.9170	4.2 b	5.2 b	0.5743
Combination	0.07 + 3.36 (1.0×)	3.2 b	3.7 b	0.6357	0.5 b	0.7 b	0.7512	0.6 ab	0.5 b	0.9170	5.3 ab	5.3 b	1.0000
Combination	0.10 + 5.04 (1.5×)	3.2 b	3.6 b	0.6482	0.7 b	0.5 b	0.7512	0.5 ab	0.4 b	0.9170	4.3 b	5.0 b	0.7079
Non-treated	0.0	17.7 a	24.8 a	**0.0001**	7.8 a	11.8 a	**0.0001**	0.9 a	1.8 a	**0.0001**	9.0 a	20.5 a	**0.0001**

a *Combination = halosulfuron + glyphosate at the specified rates. Rate relative to manufacturer's recommended rate shown parenthetically*.

b *P-values show main effects of CO_2_ level within each treatment on each data collection date. P-values are bolded where CO_2_ had a significant effect on control ratings within a treatment*.

c *Means followed by the same letter within each column and subheading are not significantly different based on Tukey's honest Significance Difference Test (P ≤ 0.05)*.

### Yellow nutsedge

Similar to results from purple nutsedge, treatments containing glyphosate or glyphosate + halosulfuron had higher control ratings than those containing halosulfuron alone at 1 week post-treatment, with the exception of glyphosate at 1.68 kg ai/ha^−1^, which was similar to halosulfuron treatments in ambient chambers and similar to halosulfuron at 0.10 kg ai/ha^−1^ in elevated chambers. CO_2_ level had no effect on control ratings at 1 week post-treatment. By 2 weeks post-treatment, yellow nutsedge control ratings were similar among all herbicide treatments in ambient chambers. In elevated chambers, yellow nutsedge treated with halosulfuron at 0.04 and 0.07 kg ai/ha^−1^ had lower control ratings than those treated with the high rate of glyphosate or any rate of the glyphosate + halosulfuron combination. There was a significant CO_2_ effect at 2 weeks post-treatment as control ratings were higher in ambient chambers when yellow nutsedge were treated with halosulfuron at 0.04 or 0.07 kg ai/ha^−1^. There was also a similar significant CO_2_ effect when glyphosate was applied at 3.36 kg ai/ha^−1^. By 3 weeks post-treatment, all herbicide treatments provided similar control and ratings ranged from 9.8 to 10 in ambient chambers and 9.3 to 10 in elevated chambers. The only treatments in which CO_2_ had an effect on control ratings were in glyphosate treatments at 1.68 and 3.36 kg ai/ha^−1^ in which control ratings were lower in elevated chambers compared with ambient.

Yellow nutsedge shoot, root, and tuber DW and tuber counts in control treatments showed that yellow nutsedge grew larger in elevated chambers (*P* = 0.0001). Similar to results observed for purple nutsedge, few differences were observed among herbicide treatments in terms of shoot, root, or tuber DW. However, in contrast to results observed in purple nutsedge in which tuber counts were similar among all treatments (including non-treated controls), in yellow nutsedge, tuber counts were significantly reduced when glyphosate was applied at either 3.36 or 5.04 kg ai/ha^−1^ and also when the combination was applied at 0.04 + 1.68 and 0.10 + 5.04 kg ai/ha^−1^ in ambient chambers. In addition, all yellow nutsedge treated with herbicide had fewer tubers than the non-treated control in elevated chambers.

## Discussion

Control ratings at 1 week post-treatment were lower in ambient and elevated chambers for both species when halosulfuron was applied alone. While both herbicides are amino acid inhibitors, their mechanism of action differs slightly. Halosulfuron is a systemic sulfonylurea herbicide that disrupts acetolactate synthase enzyme and herbicide symptoms usually begin to appear on effected weeds within 14 days, usually as a necrotic ring at the base of the plant while leaves and stems may remain green (Senseman, 2007; Anonymous, 2011). Glyphosate inhibits 5 enolpyruvylshikimate-3-phosphate synthase and symptoms (gradual yellowing, wilting, browning of shoots) are usually observable within 2–4 days on annual species and 7 days or more for perennial species (Anonymous, 2002; Senseman, 2007). Data show that while the effects of halosulfuron were slightly delayed, within 2 weeks post-treatment control ratings were generally similar among both products and the combination tank mix. Dry weights and tuber counts were also similar for most herbicide treatments at 3 weeks post-treatment.

There was no main effect for herbicide rate in either purple or yellow nutsedge regardless of CO_2_ level. Archambault et al. (2001) reported that glyphosate efficacy for control of Canada thistle (*Cirsium arvense* L.) was reduced at CO_2_ levels of 720 μmol mol^−1^ when 0.25× of the manufacturer's rate was applied compared to the 1× rate. Additionally, Ziska et al. (1999) showed decreased efficacy on common lambsquarters (*Chenopodium album* L.) under elevated CO_2_ conditions when glyphosate was applied at 0.1× compared with the label rate. Although CO_2_ concentrations used in the present study are comparable to those used in previous reports, a much higher minimum rate (0.5×) was applied in the present study which likely limited the rate and CO_2_ effect which has been observed previously with glyphosate and other herbicides. To our knowledge, there have been no earlier reports on the effect of elevated CO_2_ on halosulfuron efficacy, so we are unable compare these results with previous findings.

It is clear that both species of nutsedge grew larger in elevated CO_2_ chambers, similar to previous reports (Rogers et al., 2008). Both species had greater shoot and root DW in elevated chambers and yellow nutsedge also had greater tuber DW and tuber counts. While both species grew larger in elevated chambers, at the end of the study there were no differences in terms of weed control among any of the herbicide treated plants. Reductions in glyphosate efficacy against Canada thistle in elevated CO_2_ environments have been attributed to greater root growth and reductions in the systemic effect of the herbicide (Ziska et al., 2004). While both species of nutsedge evaluated here naturally have extensive root and tuber growth (Thullen and Keeley, 1979; Stoller and Sweet, 1987) and have been shown to have positive growth response in elevated CO_2_environments (Rogers et al., 2008), it would seem that herbicide efficacy would likely be reduced in elevated CO_2_ levels. It is likely that the rates and herbicides applied in this study were high enough to provide adequate control regardless of CO_2_ level. Further, nutsedge in this study were grown in small (3.0 L) containers, and it is possible root growth was slightly restricted, although upon visual inspection no plants appeared root-bound in the pots at the time of herbicide application.

As tubers were dried to determine biomass, it is unclear if tubers remained viable following herbicide application. Tubers also came from plants grown in ambient rather than elevated CO_2_, so any effects of elevated CO_2_ on tuber predisposition during development in the field were not captured. While both purple and yellow nutsedge have been shown to produce more tubers under elevated CO_2_ (Rogers et al., 2008), most authors attribute decreased herbicide efficacy in enriched CO_2_ environments to above ground changes including increased plant size, possible reduced stomatal conductance, less demand for aromatic amino acids (Ziska et al., 1999), or a function of high leaf starch concentrations interfering with herbicide activity (Patterson, 1993). It is unclear what effect, if any, CO_2_ concentration during tuber development has on herbicide resistance of mature nutsedge.

Elevated atmospheric CO_2_ levels evaluated in this study represent approximate concentrations predicted in year 2060 using some of the Intergovernmental Panel on Climate Change's (IPCC) more pessimistic scenarios, whereas 608 μmol mol^−1^ is never reached using more optimistic scenarios (IPCC, 2013). It is very difficult to project future CO_2_ concentrations as predictions must take into account natural and anthropogenic emissions, how the earth responds and adapts to these emissions, future socioeconomic development, and policy choices (IPCC, 2013). Further, climate models are continually improved upon and altered as more data become available. A CO_2_ concentration of 608 μmol mol^−1^ was chosen based upon some of the predicted worst-case emission scenarios. However, future research is needed to determine changes in herbicide efficacy on weed species that are more significantly impacted by CO_2_ fertilization at lower concentrations, such as C_3_ species (Leegood, 2002), to account for changes in management practices that may be needed should the more conservative climate change models play out.

Results from this study suggest that although both species of nutsedge showed a positive response to elevated CO_2_ levels (608 μmol mol^−1^), halosulfuron, glyphosate, or a combination tank mixture of the two active ingredients will provide acceptable control of both purple and yellow nutsedge at rates as low as 0.5× the manufacturer's maximum label rate when treated at a similar growth stage (i.e., 10 and 20 cm in height for purple and yellow nutsedge, respectively). Further investigation is needed to determine how herbicide efficacy would be affected by increased exposure time to elevated CO_2_ or by delaying applications until the nutsedge grew closer to its maximum size or reached the flowering stage. It would also be important to focus future research on determining the impact of herbicide application on tuber viability when applied in an enriched CO_2_ environment. While this experiment focused on a single-treatment scenario, it should be noted that both of these species typically require repeated herbicide applications supplemented with cultural practices over several years for significant tuber reduction and long-term control (Stoller et al., 1979; Keeley et al., 1983).

### Conflict of interest statement

The authors declare that the research was conducted in the absence of any commercial or financial relationships that could be construed as a potential conflict of interest.

## References

[B1] Ainsworth E. A. Long S. P. (2005). What have we learned from 15 years of free-air CO_2_ enrichment (FACE?). A meta-analytic review of the responses of photosynthesis, canopy properties and plant production to rising CO_2_. New Phytol. 165, 351–372. 10.1111/j.1469-8137.2004.01224.x 15720649

[B2] Amthor J. S. (1995). Terrestrial higher-plant response to increasing atmospheric CO_2_ in relation to the global carbon cycle. Glob. Change Biol. 1, 243–274 10.1111/j.1365-2486.1995.tb00025.x

[B3] Anonymous. (2002). RoundUp Pro® Herbicide Product Label. St. Louis, MO: M. Monsanto Co.

[B4] Anonymous. (2011). SedgeHammer® Herbicide Product Label. Yuma, AZ: Gowan Co.

[B5] Archambault D. J. Li X. Robinson D. O'Donovan J. T. Klein K. K. (2001). The Effects of Elevated CO_2_ and Temperature on Herbicide Efficacy and Weed/Crop Competition. Rept. Prairie Adapt. Res. Collab. 29. Available online at: http://www.parc.ca/pdf/research_publications/renamed/PARC-40.pdf (Accessed July 24, 2014).

[B6] Calanca P. Bolius D. Weigel A. P. Liniger M. A. (2011). Application of long-range weather forecasts to agricultural decision problems in Europe. J. Agric. Sci. Camb. 149, 15–22 10.1017/S0021859610000729

[B7] Eitzinger J. Orlandini S. Stefanski R. Naylor R. E. L. (2010). Climate change and agriculture: introductory editorial. J. Agric. Sci. Camb. 148, 499–500 10.1017/S0021859610000481

[B8] Felix J. Newberry G. (2012). Yellow nutsedge control and reduced tuber production with S-metolachlor, halosulfuron plus dicamba, and glyphosate in furrow-irrigated corn. Weed Technol. 26, 213–219 10.1614/WT-D-11-00131.1

[B9] Holm L. G. Pancho J. V. Herberger J. P. Plucknett D. L. (1991a). A Geographical Atlas of World Weeds. Malabar, FL: Krieger Publishing.

[B10] Holm L. G. Pancho J. V. Herberger J. P. Plucknett D. L. (1991b). The World's Worst Weeds: Distribution and Biology. Malabar, FL: Krieger Publishing.

[B11] IPCC. (2007). Climate change 2007: the physical science basis, in Contribution of Working Group I to the Fourth Assessment Report of the Intergovernmental Panel on Climate Change, eds Solomon S. Manning M. Chen Z. Marquis M. Averyt K. B. Tignor M. Miller H. L. (Cambridge; New York: Cambridge University Press).

[B12] IPCC. (2013). Annex II: climate system scenario tables [Prather, M., Flato, G., Friedlingstein, P., Jones, C., Lamarque, J. F., Liao, H., and Rasch, P., eds.], in Climate Change 2013: The Physical Science Basis. Contribution of Working Group I to the Fifth Assessment Report of the Intergovernmental Panel on Climate Change, eds Stocker T. F. Qin D. Plattner G. K. Tignor M. Allen S. K. Boschug J. Nauels A. Xia Y. Bex V. Midgley P. M. (Cambridge; New York: Cambridge University Press).

[B13] Keeley P. E. Thullen R. J. Miller J. H. Carter C. H. (1983). Comparison of six cropping systems for yellow nutsedge (*Cyperus esculentus*) control. Weed Sci. 31, 63–67.

[B14] Keeling R. F. Piper S. C. Bollenbacher A. F. Walker J. S. (2009). Atmospheric CO_2_ records from sites in the SIO air sampling network, in Trends: A Compendium of Data on Global Change. Carbon Dioxide Information Analysis Center, Oak Ridge National Laboratory (Oak Ridge, TN: U.S. Department of Energy). 10.3334/CDIAC/atg.035

[B15] Kimball B. A. (1983). Carbon Dioxide and Agricultural Yield: an Assemblage and Analysis of 770 Prior Observations. USDA/ARS Water Conservation Laboratory Report Number 14. Phoenix, AZ: US Department of Agriculture, Agricultural Research Service.

[B16] Leegood R. C. (2002). C_4_ photosynthesis: principles of CO_2_ concentration and prospects for its introduction into C_3_ plants. J. Exp. Bot. 53, 581–590. 10.1093/jexbot/53.369.581 11886878

[B17] Littell R. C. Milliken G. A. Stroup W. W. Wolfinger R. D. (1996). SAS System for Mixed Models. Cary, NC: SAS Institute.

[B18] Manea A. Leishman M. R. Downey P. O. (2011). Exotic C_4_ grasses have increased tolerance to glyphosate under elevated carbon dioxide. Weed Sci. 59, 28–36 10.1614/WS-D-10-00080.1

[B19] McCloskey W. B. (2004). Nutsedge (*Cyperus* spp.) control in alfalfa, in Proceedings of the National Alfalfa Symposium (San Diego, CA), 13–15.

[B20] Mitchell R. J. Runion G. B. Prior S. A. Rogers H. H. Amthor J. S. Henning F. P. (1995). Effects of nitrogen on *Pinus palustris* foliar respirator responses to elevated atmospheric CO2 concentration. J. Exp. Bot. 46, 1561–1567 10.1093/jxb/46.10.1561

[B21] Oerke E. C. (2006). Crop losses to pests. J. Agric. Sci. Camb. 144, 31–43 10.1017/S0021859605005708

[B22] Patterson D. T. (1993). Implications of global climate change for impact of weeds, insects and plant diseases, in International Crop Science, ed Buxton D. R. (Madison, WI: Crop Sci. Soc. Am.), 273–280.

[B23] Patterson D. T. (1995). Weeds in a changing climate. Weed Sci. 43, 685–701.

[B24] Patterson D. T. Flint E. P. (1980). Potential effects of global atmospheric CO_2_ enrichment on the growth and competitiveness of C_3_ and C_4_ weed and crop plants. Weed Sci. 28, 71–75.

[B25] Patterson D. T. Flint E. P. (1990). Implications of increasing carbon dioxide and climate change for plant communities and competition in natural and managed ecosystems, in Impact of Carbon Dioxide, Trace Gases, and Climate Change on Global Agriculture ASA Special Publication No. 53, eds Kimball B. A. Rosenberg N. J. Allen L. H. Jr. (Madison, WI: American Society of Agronomy), 83–110.

[B26] Rogers H. H. Dahlman R. C. (1993). Crop responses to CO_2_ enrichment. Vegetatio 104/105, 117–131 10.1007/BF00048148

[B27] Rogers H. H. Heck W. W. Heagle A. S. (1983). A field technique for the study of plant response to elevated carbon dioxide concentrations. J. Air Pollut. Control Assn. 33, 42–44 10.1080/00022470.1983.10465546

[B28] Rogers H. H. Runion G. B. Prior S. A. Price A. J. Torbert H. A. (2008). Effects of elevated atmospheric CO_2_ on invasive plants: comparison of purple and yellow nutsedge (*Cyperus rotundus* L. and *C. esculentus* L.). J. Environ. Qual. 37, 395–400. 10.2134/jeq2007.0155 18268302

[B29] Santos B. M. Morales-Payan J. P. Stall W. M. Bewick T. A. Shilling D. G. (1997). Effect of shading on the growth of nutsedges (*Cyperus* spp.). Weed Sci. 45, 670–673.

[B29a] Senseman S. A. (ed). (2007). Herbicide Handbook, 9th Edn. Champaign, IL: Weed Science Society of America, 458.

[B30] Steckel L. (2007). Yellow Nutsedge. UT Extension, Univ. of Tennessee. Available online at: http://www.utextension.utk.edu/publications/wfiles/W136.pdf (Accessed July 24, 2014).

[B31] Stoller E. W. Sweet R. D. (1987). Biology and life cycle of purple and yellow nutsedges (*Cyperus rotundus* and *C. esculentus*). Weed Technol. 1, 66–73.

[B32] Stoller E. W. Max L. M. Slife F. W. (1979). Yellow nutsedge (*Cyperus esculentus*) competition and control in corn (*Zea mays*). Weed Sci. 27, 32–37.

[B33] Thullen R. J. Keeley P. E. (1979). Seed production and germination in *Cyperus esculentus* and *C. rotundus*. Weed Sci. 27, 502–505.

[B34] USDA-ARS. (1970). Selected Weeds of the United States. Agriculture Handbook 366. Washington, DC: USDA.

[B35] Wills G. D. (1987). Description of purple and yellow nutsedge (*Cyperus rotundus* and *C. esculentus*). Weed Technol. 1, 2–9.

[B36] Ziska L. H. (2002). Influence of rising atmospheric CO_2_ since 1900 on early growth and photosynthetic response of a noxious invasive weed, Canada thistle (*Cirsium arvense*). Funct. Plant Biol. 29, 1387–1392 10.1071/FP02052 32688738

[B37] Ziska L. H. Runion G. B. (2007). Future weed, pest and disease problems for plants, in Agroecosystems in a Changing Climate, eds Newton P. C. D. Carran R. A. Edwards G. R. Niklaus P. A. (Boca Raton, FL: CRC Press), 261–287.

[B38] Ziska L. H. Teasdale J. R. (2000). Sustained growth and increased tolerance to glyphosate observed in a C_3_ perennial weed, quackgrass (*Elytrigia repens*), grown at elevated carbon dioxide. Funct. Plant Biol. 27, 159–166 10.1071/PP99099

[B39] Ziska L. H. Faulkner S. S. Lydon J. (2004). Changes in biomass and root:shoot ratio of field-grown Canada thistle (*Cirsium arvense*), a noxious, invasive weed, with elevated CO_2_: implications for control with glyphosate. Weed Sci. 52, 584–588 10.1614/WS-03-161R

[B40] Ziska L. H. Sicher R. C. George K. Mohan J. E. (2007). Rising atmospheric carbon dioxide and potential impacts on the growth and toxicity of poison ivy (*Toxiocodendron radicans*). Weed Sci. 55, 288–292 10.1614/WS-06-190

[B41] Ziska L. H. Teasdale J. R. Bunce J. A. (1999). Future atmospheric carbon dioxide may increase tolerance to glyphosate. Weed Sci. 47, 608–615 10.1093/jxb/46.10.1561

